# An explorative approach of the InovSafeCare model for education in infection prevention and control: a cross-sectional study among undergraduate nursing students in sub-Saharan Africa

**DOI:** 10.1080/16549716.2026.2728504

**Published:** 2026-09-15

**Authors:** Filipa Ventura, Pedro Parreira, Gisela Mosca Teixeira, Pedro Lucas, Paulo Lino Kidayi, Harilalaina Willy Franck Randriamarotia, Rivo Solotiana Rakotomalala, Eyeshope J. Dausen, Ewelina Chawłowska, João Graveto, Márcia Pestana-Santos, Paulo Santos-Costa, Jane Januarius Rogathi, Christina Chuki Mtuya, Lova Narindra Randriamanantsoa, Guy Emmanuel Raoelison, Liliane Ravelonarivo, Zo Lalaina Ony Andriamalala, Stéphanie Norotiana Andriamiharisoa, Narindrarimanana Avisoa Randriamihangy, Hobiherinjato Jhonse Tovohandraina, Vololomboahanginirina Ella Françoise Andriamampiandra, Rose M. Laisser, Livuka Nsemwa, Marlena Szewczyczak, Edyta Cudak-Kasprzak, Krystyna Jaracz, Grażyna Bączyk, João Agrelos, Carla Nascimento, M. Rosário Pinto

**Affiliations:** a Nursing School of the University of Coimbra (ESEUC), Health Sciences Research Unit: Nursing (UICISA E), University of Coimbra, Coimbra, Portugal; b The Nursing Research, Innovation and Development Centre of Lisbon (CIDNUR), Lisbon, Portugal; c School of Nursing, KCMC University, Moshi, Tanzania; d Faculty of Medicine, University of Antananarivo, Antananarivo, Madagascar; e Faculty of Medicine, University of Mahajanga, Mahajanga, Madagascar; f Archbishop Anthony Mayala School of Nursing (AAMSoN), Catholic University of Health and Allied Sciences, Mwanza, Tanzania; g Department of Nursing, Poznan University of Medical Sciences, Poznań, Poland; h Department of Anaesthesiological and Intensive Care Nursing, University of Medical Sciences, Poznań, Poland; i Department of Preventive Medicine, Poznan University of Medical Sciences, Poznań, Poland; j Institut de Formation et de Recherches Paramédicales, University of Antananarivo, Antananarivo, Madagascar; k School of Nursing, University of Mahajanga, Mahajanga, Madagascar; l Local Unit of the Programme for the Prevention and Control of Infections and Antimicrobial Resistance (UL-PPCIRA), Western Lisbon Local Health Unit (ULSLO), Lisbon, Portugal

**Keywords:** Competency-based education, infection control, nursing education, self efficacy, skepticism

## Abstract

**Background:**

Adherence to healthcare-associated infection prevention and control best practices is essential to reducing morbidity and mortality in sub-Saharan Africa, where structural and educational challenges may affect adherence. Strengthening nursing education requires contextually appropriate frameworks and valid tools to evaluate them.

**Objective:**

To evaluate the psychometric properties of the culturally adapted InovSafeCare Questionnaire and examine the structural relationships proposed by the InovSafeCare Model among undergraduate nursing students in sub-Saharan Africa.

**Methods:**

A multiple-method approach was employed, involving the translation, cultural adaptation, and psychometric evaluation of the InovSafeCare Questionnaire. Data were collected from 486 undergraduate nursing students across four higher education institutions in two sub-Saharan African countries. Principal component analysis and reliability testing examined the questionnaire structure, followed by path analysis to evaluate the relationships among the model constructs.

**Results:**

Psychometric analysis yielded a 10-factor solution explaining 52.1% of the variance, with Cronbach’s alpha value ranging from 0.64 to 0.88. The structural model demonstrated acceptable-to-moderate fit (χ^2^/df = 5.59; CFI = 0.88; GFI = 0.95; RMSEA = 0.097). No direct relationships were found between educational constructs and self-reported adherence. Instead, these associations operated through psychological and contextual mediators, including motivation, expectations, skepticism, environmental complexity, and overload and fatigue. The model explained 18.9% of the variance in adherence.

**Conclusions:**

The culturally adapted InovSafeCare Questionnaire demonstrated acceptable psychometric properties, and structural analysis provided preliminary support for the model’s applicability in the sub-Saharan African sample. Further research should replicate the measurement and structural model across settings before considering its use to inform contextually adapted educational interventions.

## Paper Context

- **Main findings**: Psychological and contextual factors, such as motivation, expectations, skepticism, and fatigue significantly mediate the relationship between education and adherence to infection prevention practices among nursing students in sub-Saharan Africa.
- **Added knowledge**: This study reveals that educational interventions alone are insufficient to promote adherence unless reinforced by supportive institutional environments and aligned with students’ clinical realities.
- **Global health impact for policy and action**: Strengthening infection prevention requires policy and practice reforms that integrate experiential learning, reduce institutional skepticism, and address student workload to improve adherence and patient safety globally.

## Background

Healthcare-associated infections (HAIs) remain a major public health concern, particularly in sub-Saharan Africa, where limited resources, workforce shortages, and infrastructure constraints hinder effective healthcare-associated infection prevention and control (HAI-PC) [1–5]. Adherence to HAI-PC best practices is essential to reducing preventable morbidity and mortality. However, adherence is influenced not only by knowledge and technical competence but also by behavioural, organizational, and environmental factors that shape how infection prevention practices are understood and enacted in clinical settings [1,6,7].

Nursing education is therefore an important component of infection prevention. International bodies, including the World Health Organization and the International Council of Nurses, emphasize structured, competency-based education to equip future nurses with the knowledge, skills, and behaviours required for safe practice [8–10]. The WHO guidance further highlights the importance of multimodal approaches that combine education with system change, monitoring and feedback, communication, and safety culture [11,12]. Against this background, the InovSafeCare Model offers a potentially useful baseline framework for future regionally adapted HAI-PC education because it integrates educational experiences with psychological and contextual factors that may influence adherence [13–15]. As the model and its associated questionnaire were originally developed and evaluated in European nursing education settings, however, their applicability cannot be assumed in sub-Saharan African contexts.

Accordingly, this study had two sequential objectives: (1) to translate and culturally adapt the InovSafeCare Questionnaire and evaluate its psychometric structure and internal consistency among undergraduate students in participating sub-Saharan African higher education institutions and (2) to examine the structural relationships among the empirically derived constructs to assess the applicability of the InovSafeCare Model in this context.

### The InovSafeCare model

The InovSafeCare Model was developed through a collaborative European initiative to support HAI-PC education in nursing programmes [13–15]. Its development was informed by literature review, expert consultation, cultural adaptation across participating European settings, and exploratory application among nursing students. The model integrates competency-based education, simulation-based learning, reflective practice, and psychological and contextual determinants relevant to HAI-PC adherence [13–15]. Constructs such as self-efficacy, motivation, expectations, skepticism, and perceived environmental complexity are included because educational and behavioural evidence suggests that adherence to infection prevention practices are shaped by more than educational exposure alone [6,7,13,14].

The model also led to the development of the InovSafeCare Questionnaire, which assesses students’ perceptions, competencies, learning experiences and adherence-related constructs [13]. Previous applications in European settings provided initial evidence regarding its measurement properties and the relationships among its theoretical dimensions [13–15]. Nevertheless, instruments developed in one cultural and educational context may not retain the same empirical structure when transferred to another [16]. This is particularly relevant in sub-Saharan Africa, where educational conditions, clinical learning environments, organizational resources, and healthcare-system characteristics may differ substantially from those for which the original model was developed [1,7].

For this reason, the psychometric evaluation of the culturally adapted questionnaire represents a necessary first step in examining the transferability of the InovSafeCare framework. Establishing whether the questionnaire retains a coherent and internally consistent structure provides the basis for interpreting its constructs in the new context. Once this measurement structure is established, the relationships among the resulting constructs can then be examined to determine whether the theoretical pathways proposed by the InovSafeCare Model are supported in sub-Saharan African settings.

This sequential evaluation is relevant because the value of the InovSafeCare Model as a baseline for future regionally adapted education depends not only on whether its constructs can be meaningfully measured but also on whether the relationships proposed among educational, psychological, contextual, and adherence-related dimensions remain empirically plausible across different cultural and health-care environments. Examining these relationships therefore provides an initial basis for assessing the model’s theoretical applicability in sub-Saharan Africa, and for informing subsequent replication and adaptation research.

## Methods

This cross-sectional observational study comprised two sequential empirical components. First, the InovSafeCare Questionnaire underwent translation, cultural adaptation, and psychometric evaluation to examine its measurement structure in the participating sub-Saharan African settings. Second, following establishment of the empirically derived structure, path analysis was used to examine the structural relationships among the constructs and assess the applicability of the InovSafeCare Model in this context.

### Participants and data collection

Data were collected in November 2023 from undergraduate students enrolled at four participating HEIs in Tanzania and Madagascar. Students were eligible if they were aged 18 years or older and enrolled in an undergraduate nursing, midwifery, or anaesthesia programme, regardless of previous higher education experience. A census sampling approach was adopted within each institution, whereby all eligible students present during the scheduled data collection sessions were invited to participate. The final sample comprised 486 students and was considered adequate for factor analysis and structural modelling based on the recommended sample-size guidance for multivariate analysis [17].

The InovSafeCare Questionnaire consisted of 93 items rated on a five-point Likert scale, ranging from 1 (strong disagreement) to 5 (strong agreement), and evaluates 14 constructs related to healthcare-associated infections. Each construct is assessed through a subset of items, with individual construct scores determined by averaging the responses of the respective items. Higher scores indicate stronger agreement with the measured construct, reflecting students’ perceptions and self-reported adherence to best practices in the HAI-PC. These constructs include theoretical context, formative approach, learning and teaching approaches, learning sources and context, clinical best practices, best practices culture, attitudes, skepticism, motivation, environmental complexity, best practice deviation, expectations, and overload and fatigue [13,14]. Socio-demographic and academic variables, including year of study, programme, and previous educational background, also collected. To minimize social desirability and perceived coercion, the questionnaires were self-administered without faculty supervision, and participants were informed that their responses would not influence academic evaluation.

### Translation and cultural adaptation

Translation and cultural adaptation follow International Test Commission [16] guidance for adapting instruments across cultural and linguistic settings [16]. Forward translation was conducted by bilingual experts fluent in the source and target languages, followed by reconciliation of discrepancies by an expert panel. Independent back translation into the original language was subsequently undertaken to identify inconsistencies and assess conceptual equivalence. Subject-matter experts then reviewed the translated versions for accuracy, clarity, and cultural appropriateness. Cognitive debriefing with students from the target population was used to identify ambiguities or culturally sensitive content, after which the translated versions were finalized for implementation.

### Psychometric evaluation

Data were analysed using SPSS version 24.0. The dataset was first examined using descriptive statistics and screened for completeness and consistency. Construct validity was explored by examining item-total correlations and the empirical dimensional structure of the culturally adapted questionnaire. Item-total correlations above 0.40 were considered indicative of adequate alignment between an item and its corresponding construct.

Sampling adequacy and factorability were evaluated using the Kaiser–Meyer–Olkin (KMO) measure and Bartlett’s test of sphericity. Principal component analysis (PCA) with Varimax rotation was used as an exploratory data-reduction approach to examine the empirical structure of the culturally adapted questionnaire. Component retention was guided by eigenvalues greater than 1, inspection of the scree plot, item loadings, absence of problematic cross-loadings, and interpretability of the resulting structure [18]. Items with loadings below 0.40 or problematic cross-loadings were considered for removal. Internal consistency of the retained dimensions was evaluated using Cronbach’s alpha [19].

### Model verification

Following establishment of the empirically derived questionnaire structure, path analysis was conducted using AMOS to examine the direct and indirect relationships among the retained constructs and evaluate the structural relationships proposed by the InovSafeCare Model. The model was estimated using the maximum likelihood method. Given the exploratory purpose of the structural evaluation, no a priori directional hypotheses were specified. Prior to structural modelling, distributional characteristics and construct intercorrelation were examined. Univariate normality was assessed using skewness and kurtosis, with values of |Sk| <3 and |Ku| <7 considered indicative of no severe departure from normality. Construct correlations were also inspected as a preliminary screening for excessive overlap among variables. Model fit was evaluated using the chi-square/degrees-of-freedom ratio (χ^2^/df), Comparative Fit Index (CFI), Goodness-of-Fit Index (GFI), and Root Mean Square Error of Approximation (RMSEA). These indices were interpreted collectively when evaluating the adequacy of the structural model. Only statistically significant pathways (*p* ≤ 0.05) were retained in the final presented model.

### Ethical considerations

The study was conducted in accordance with the Declaration of Helsinki. Participation was voluntary, and written informed consent was obtained from all participants prior to data collection. Participants received detailed information about the study objectives, procedures, potential risks, and their right to withdraw at any time without consequences. Confidentiality and anonymity were ensured through coded and anonymized data handling, and the data were used exclusively for research purposes.

## Results

### Participant characteristics

The psychometric and structural analyses included data from 486 students enrolled across the four participating higher education institutions. The response rate could not be determined because the total number of eligible students present during the data collection was not systematically recorded. Prior to analysis, the dataset was screened for completeness and consistency using frequency distribution and descriptive statistics. No missing values were identified, therefore, all analyses were conducted using complete-case data.

The sample comprised 67.3% female and 32.7% male students and was evenly distributed between Tanzania and Madagascar. Regarding academic programmes, 69.1% were enrolled in nursing, 19.3% in midwifery and 11.5% in anaesthesia. Students were distributed across the first (8.8%), second (39.7%), third (35.2%), and fourth (16.3%) years of study. The mean age was 23.18 years (SD = 2.25).

### Translation and cultural adaptation

The translation and cultural adaptation of the questionnaire involved six independent translators, three of them Kiswahili speakers who had fluent English and three conversant in English and French. Two bilingual experts did the first phase, fluent in both the source and target languages, ensuring the maintenance of the original meaning, while domain-specific terminology was accurately translated. A second stage focused on discussing the discrepancies between the translated versions, involved a panel of 19 experts, four with deep knowledge of the conceptual, semantic, and functional dimensions of the InovSafeCare Questionnaire, and the others were experts in HAI-PC: six English speakers and nine bilingual experts from SSA (five in Kiswahili and four in French). A third independent translator expert in Kiswahili and French, who had no prior knowledge of the instrument, performed the back translation into the original language. The comparison between the original and back-translated versions, which was done by a panel of four subject matter experts (two bilinguals in Kiswahili/English and two in English/French), allowed the review of the translations for accuracy and cultural appropriateness.

Six students and four lecturers from the target population (three fluent in Kiswahili and three in French) participated in the cognitive debriefing. No ambiguities or culturally sensitive items were pointed out. These were the final versions applied.

### Psychometric evaluation

Initial exploratory analysis produced a diffuse factor solution. Following iterative examination of factor loadings, cross-loadings, item performance, theoretical interpretability, and parsimony, the final 10-factor solution was retained, explaining 52.1% of the variance. Sampling adequacy was satisfactory (KMO = 0.843), and Bartlett’s test of sphericity was significant (χ^2^ = 17,906.981, *p* < .001), supporting the factorability of the data.

The final empirically derived structure comprised theoretical context, training and culture of best practices, adherence to best practices, extrinsic motivation, intrinsic motivation, self-efficacy, environmental complexity, expectations, skepticism, and overload and fatigue (Table 1).

**Table 1.** Empirically-driven structure of InovSafeCare for sub-Saharan countries.

Dimensions and Items
Theoretical context
1. Chain of infection.
2. Individual assessment of the risk of infection during patient admission and patient placement/isolation.
3. Hand hygiene.
4. Respiratory tract infection.
5. Use of personal protective equipment.
6. Decontamination of medical/clinical devices and equipment.
7. Decontamination of environmental surfaces in hospitals.
8. Safe use of protective clothing.
9. Proper clinical waste management.
10. Safe preparation and administration of intravenous medications.
11. Prevention of exposure to microbial agents in the workplace.
12. Additional precautions for specific transmission routes (e.g. droplet transmission, …).
Training and Culture of Best Practices
1. There are mandatory courses dedicated to the development of competencies.
2. HCAI prevention and control topics are included in theoretical classes.
3. Students are offered workshops or classes by professionals (e.g. from practice).
4. Visual resources and other technologies (e.g. apps, …) are used in classrooms to explain HCAI topics.
5. Teachers give feedback to the students about their development.
6. Students receive guidance from supervisors on adequate HCAI prevention and control measures.
7. Supervisors make sure that we use the personal protective equipment available.
8. If students do not follow HCAI prevention and control measures, they receive feedback to improve their performance.
9. The nursing team motivates students to follow HCAI prevention and control measures.
10. In an HCAI-dedicated course.
11. In other general courses (e.g. pediatric nursing, elderly care nursing).
12. From the ward nurses during clinical placement/practice.
13. From the school’s supervisors during clinical placement/practice.
14. Hand hygiene is promoted in the clinical area
Adherence to Best Practices
1. I have forgotten to follow safety protocols when required.
2. I have worn accessories below the elbows (e.g. wrist watches, bracelets, rings,).
3. I have not used gloves when required.
4. I have not washed and/or disinfected my hands when required.
5. I have not worn face masks when required.
6. I have assisted a patient without changing my gloves, masks or other protective equipment.
Extrinsic motivation
1. I am driven by professional ethics.
2. I will get better grades.
3. Other team members would think better of me.
4. Other team members also follow them.
5. I will meet the expectations of my supervisor.
Intrinsic motivation
1. My main drive is patient health.
2. I understand its implications.
3. I aim for quality care.
4. I am concerned about my patients’ safety.
5. I am concerned about my own safety.
Self-efficacy
1. Even if others did not, I would still follow the safety measures.
2. I am confident that I could deal efficiently with HCAI prevention and control measures under unexpected events (e.g. unexpected patient transfer).
3. I am confident that I could deal efficiently with HCAI prevention and control measures under stressful events (e.g. performing a nursing procedure for the first time).
Environmental complexity
1. Disorganized rooms or units.
2. Too many patients in the same room/space.
3. A significant number of requests from patients and/or family members.
4. Talking with peers.
5. Caring for patients with multiple conditions.
6. Emotionally demanding cases.
7. Having to perform multiple clinical tasks.
Expectations
1. I am satisfied with my career choice.
2. My clinical experiences keep me motivated into pursuing a career as a professional.
3. I am very interested in continuing a career as a healthcare professional.
4. Being a healthcare professional will fulfill my professional expectations.
Skepticism
1. Some HCAI safety protocols are unnecessary.
2. I express my reservations to the application of HCAI prevention and control measures that I deem unnecessary.
3. The use of so much disposable material is unnecessary.
4. Personal protective equipment is uncomfortable.
5. I feel upset if my supervisor is constantly monitoring my HCAI prevention and control measures.
Overload & Fatigue
1. I have experienced academic overload.
2. I have experienced difficult moments in my personal life.
3. Clinical placements/practice have coincided with periods when I was feeling tired.
4. I have felt emotionally tired.
5. I have felt mentally tired.
6. I have felt physically tired.

Six dimensions retained close correspondence with constructs from the original theoretical structure: theoretical context, environmental complexity, adherence to best practices, overload and fatigue, expectations, and skepticism. The original motivation construct was separated into two interpretable dimensions, identified as Intrinsic Motivation and Extrinsic Motivation. The Self-Efficacy dimension retained three items while items relating to formative approaches and best-practice culture were represented within the empirically derived Training and Culture of Best Practices dimension.

Internal consistency coefficients ranged from α = 0.64 to α = 0.88, indicating variable but generally acceptable reliability across the retained dimensions. Dimension-specific estimates, standardized regression weights, reliability coefficients, and goodness-of-fit statistics are presented in Supplementary File I.

### Model verification

Following establishment of the empirically derived measurement structure, a path analysis was conducted to examine the structural relationships among the retained constructs. Skewness and kurtosis values indicated no severe departures from univariate normality (|Sk| <3; |Ku| <7), and inspection of construct intercorrelation did not indicate extreme pairwise associations.

**Figure 1.**
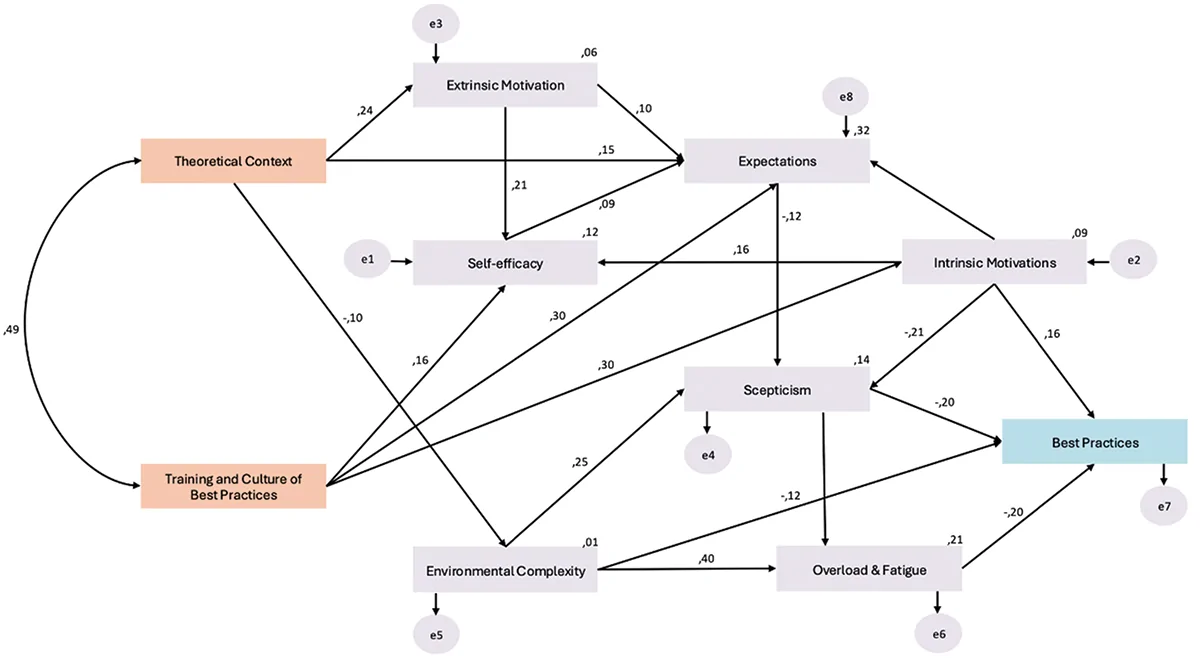
Path analysis.

The final structural model demonstrated moderate fit to the data (χ^2^/df = 5.59; CFI = 0.88; GFI = 0.95; RMSEA = 0.097). Only statistically significant pathways (*p* ≤ 0.05) were retained in the presented model (Figure 1).

No direct pathways were observed between the two educational constructs, i.e. theoretical context and training and culture of best practices, and self-reported adherence. instead, their associations with adherence occurred through psychological and contextual constructs.

Theoretical context showed a small total association with adherence (β = 0.01), operating through pathways involving extrinsic motivation, expectations, skepticism, environmental complexity, and overload and fatigue. Skepticism had a negative direct association with adherence (β = −0.20), while Environmental Complexity was positively associated with Overload and Fatigue (β = 0.40), which was in turn negatively associated with adherence.

Training and Culture of Best Practices showed a total association with adherence of β = 0.07. Its indirect pathways involve self-efficacy, intrinsic motivation, expectations, and skepticism. Intrinsic Motivation showed a positive direct association with adherence (β = 0.16), whereas Skepticism again showed a negative association (β = −0.20).

Self-Efficacy did not show a direct pathway to adherence but was associated with intrinsic motivation (β = 0.13; *p* < 0.05), extrinsic motivation (β = 0.21; *p* < 0.05), and skepticism (β = −0.11; *p* < 0.05). Overall, the structural model explained 18.9% of the variance in self-reported adherence (R^2^ = 0.189).

## Discussion

This study examined the applicability of the InovSafeCare framework in participating sub-Saharan African higher education settings through two sequential objectives: psychometric evaluation of the culturally adapted questionnaire and examination of the structural relationships among the resulting constructs. The findings provide preliminary support for both components while also indicating some context-related differences. Psychometric analysis resulted in a 10-dimension empirical structure with generally acceptable internal consistency, while structural analysis showed that the educational constructs were associated with self-reported adherence through indirect rather than direct pathways. Together, these findings suggest that core elements of the InovSafeCare framework remain empirically recognizable in this context, although neither its measurement structure nor its structural relationships were reproduced identically in the European setting.

The psychometric findings provide the first indication of this balance between consistency and contextual variation. The final 10-dimension structure explained 52.1% of the variance, with internal consistency coefficients ranging from α = 0.64 to 0.88. Several dimensions retained close conceptual correspondence with the original framework, including theoretical context, environmental complexity, adherence to best practices, expectations, skepticism, and overload and fatigue. Other educational and motivational items were reorganized within the empirical solution, resulting in a more consolidated structure than the 14 dimensions reported in the original European validations [13,14]. The more parsimonious structure does not necessarily imply a loss of theoretical relevance, but it may rather indicate that some dimensions that were empirically distinct in the original European samples were less clearly differentiated in the participating sub-Saharan African settings, underscoring the importance of reassessing measurement structure when instruments are transferred across cultural and educational contexts [16]. At the same time, the lower internal consistency observed for some dimensions, particularly at the lower end of the α range, supports interpreting the adapted measurement structure as preliminary and in need of further replication [16,19].

The second objective concerned whether the structural relationships among the empirically derived constructs supported the theoretical organization of the InovSafeCare Model. No direct pathways were identified between Theoretical Context or Training and Culture of Best Practices and self-reported adherence. Instead, their associations occurred through psychological and contextual constructs, including motivation, expectations, skepticism, environmental complexity, self-efficacy, and overload and fatigue. The theoretical significance of this finding lies in the distinction between educational exposure itself and the mechanisms through which educational experiences are related to self-reported practice. Within the structure identified in this sample, educational constructs were positioned upstream of psychological and contextual factors rather than directly adjacent to adherence.

This general pattern shows some consistency with the earlier European application of the InovSafeCare framework. In the Portuguese path model, higher-education and clinical-setting dimensions also operate substantially through mediating variables, including motivation, fatigue, personal attitudes, and other individual-level constructs [14]. Thus, across both settings, the relationship between educational context and self-reported HAI-PC practices appears to involve intermediate psychological or experiential mechanisms rather than a simple direct association. The recurrence of motivational constructs and fatigue-related factors across settings provides further support for their theoretical relevance within the broader framework [13,14].

However, important differences were also observed. In the Portuguese model, self-efficacy showed a significant direct association with self-reported HAI-PC practices, alongside intrinsic motives, personal objections to HAI-PC measures, and distraction [14]. In the present model, self-efficacy did not have a direct pathway to adherence; instead, it was associated with intrinsic and extrinsic motivation and negatively associated with skepticism. Intrinsic motivation retained a positive direct association with adherence, whereas skepticism showed the strongest negative association. The present model also gives greater prominence to expectations and environmental complexity within the indirect pathways. These differences suggest that, although some underlying mechanisms show consistency across settings, the way in which they are organized within the structural model may be context sensitive.

Skepticism warrants particular theoretical attention in this respect. The construct is conceptually related to the personal objection dimension included in the original European framework, which captured reservations and discomfort concerning HAI-PC practices [13,14]. The negative association between skepticism and self-reported adherence in the present study is therefore broadly consistent with the theoretical expectation that reservations regarding infection-prevention measures are related to reported practice. However, its recurrent position across several indirect pathways in the present model suggests that this construct may occupy a more prominent structural role in our sample. This should not be interpreted as evidence that skepticism constitutes a causal or modifiable practice deficit, but rather as an indication that the relationship between educational experiences and adherence-related perceptions may be partly organized around students’ appraisal of the credibility or acceptability of HAI-PC measures.

Overload and fatigue also remained theoretically relevant across settings. Fatigue formed part of the original InovSafeCare framework and contributed to the Portuguese structural model [13,14]. In the present analysis, overload and fatigue appeared within indirect pathways linking environmental complexity and skepticism to adherence. Its recurrence across culturally and institutionally different samples supports retaining workload-related experiences as part of the theoretical framework, while the specific pathways through which they operate may vary according to educational and clinical context.

The explanatory performance of the present structural model nevertheless requires some caution. The model explained 18.9% of the variance in self-reported adherence, compared with approximately 22% in the previous Portuguese application [14]. Moreover, the present model demonstrated a moderate fit (χ^2^/df = 5.59; CFI = 0.88; GFI = 0.95; RMSEA = 0.097). These results indicate that the proposed relationships provide only a partial representation of the adherence-related processes in this sample. The absence of direct educational pathways may therefore reflect the genuinely mediated nature of the theoretical relationships, but it may also be influenced by measurement characteristics, contextual variables not represented in the model, or differences in the way constructs are organized following cultural adaptation [13,14]. The findings should consequently be interpreted as exploratory evidence regarding the model’s structural applicability, rather than as confirmation of a definitive explanatory model.

Several limitations should be considered. First, the cross-sectional design establishes associations among constructs but does not permit causal or temporal interpretation of the observed pathways. Second, adherence was self-reported and may therefore differ from behaviour observed in clinical practice. Future validation could combine self-report measures with objective or observational indicators. Third, although the study involved four higher education institutions in two countries, educational and healthcare-system diversities across sub-Saharan Africa limits broader generalization. The inability to calculate a response rate also means that non-response bias cannot be excluded. Finally, PCA was used as an exploratory data-reduction approach during the cultural adaptation process. Although appropriate for examining the empirical organization of the adapted item set, common factor extraction and confirmatory factor analysis in independent samples would provide stronger evidence regarding latent construct validity and the stability of the 10-dimension structure.

### Future application and research directions

The present study was designed to examine the measurement and theoretical applicability of the InovSafeCare framework and should not be interpreted as an applied needs assessment or a programme-planning study. Accordingly, the observed structural pathways do not establish skepticism, fatigue, motivation, or other mediating constructs as causal intervention targets. Rather, they provide preliminary information about which theoretical relationships warrant further investigation as the framework is replicated and considered for regional adaptation.

Future research should first seek to replicate the 10-dimension measurement structure and structural relationships in independent samples and across additional sub-Saharan African educational and health-care settings. Longitudinal and intervention-based designs would then be required to determine whether changes in specific psychological or contextual constructs precede changes in adherence-related outcomes. For example, because skepticism and overload and fatigue occupied recurrent positions within the present structural model, future studies could examine whether educational approaches addressing understanding and trust in HAI-PC recommendations or organizational approaches addressing workload-related experiences, modify these relationships. Similarly, the indirect role of educational constructs suggests that subsequent research could explore how academic preparation interacts with clinical learning environments before determining whether adaptations to curricula or placement-based education are warranted. In this sense, the present findings provide a theoretical and measurement baseline for subsequent adaptation research rather than direct evidence for specific educational or organizational interventions.

## Conclusion

The culturally adapted InovSafeCare Questionnaire demonstrated acceptable construct validity and internal consistency across a 10-dimension empirical structure in a multi-site sub-Saharan African sample. Structural path analysis provided preliminary support for the applicability of the InovSafeCare Model, showing that educational constructs were associated with self-reported adherence indirectly through psychological and contextual mediators, notably intrinsic motivation, skepticism, and overload and fatigue. The model explained 18.9% of the variance in adherence and demonstrated moderate fit, indicating partial rather than definitive support for the proposed structural relationships. These findings provide a preliminary measurement and theoretical basis for further replication across settings. Future longitudinal and intervention-based research is needed to determine the stability of the identified pathways and their potential relevance to subsequent regional adaptation of HAI-PC education.

## Supplementary Material

STROBEchecklistv4_GlobalHealthAction.docx

Supplementary file 1.docx

## Data Availability

The datasets used and/or analyzed during the current study are available from the corresponding author upon reasonable request.
