# Supplementary material for: An explorative approach of the InovSafeCare model for education in infection prevention and control: a cross-sectional study among undergraduate nursing students in sub-Saharan Africa
Source: Glob Health Action. 2026 Sep 15;19(1):2728504. doi: 10.1080/16549716.2026.2728504 (PMC13580379; doi:10.1080/16549716.2026.2728504)
Supplement: Supplementary file 1 [file ZGHA_A_2728504_SM8057.docx]

**Supplementary file I**

Confirmatory structural analysis of Dimentions: Estimates, Standard-errors (SE), Critical ratios (CR), and Standardized regression weights (SRW): Means (M), Standard Deviations (SD), *Cronbach’ s Alpha* and goodness of fit indexes (χ2/df , *NFI, CFI, RMSEA and* RMSEA 90% Confidence Interval).

| **Dimensions** | **Items** | **Estimate** | **SE** | **CR** | ***SRW*** | ***M*** | ***SD*** | *Cronbach’ s Alpha* | **χ2/df** | ***NFI*** | ***CFI*** | ***RMSEA*** | **RMSEA**  **90% Confidence Interval**  **LO 90 HI 90** |
| --- | --- | --- | --- | --- | --- | --- | --- | --- | --- | --- | --- | --- | --- |
| Theoretical context | TCont1  TCont2  TCont3  TCont4  TCont5  TCont6  TCont7  TCont8  TCont9  TCont10  TCont11  TCont12 | 0,467  0,456  0,608  0,544  0,623  0,592  0,579  0,745  0,47  0,588  0,456  0,374 | 0,045  0,043  0,035  0,033  0,038  0,032  0,033  0,045  0,03  0,036  0,041  0,045 | 10,315  10,684  17,569  16,565  16,535  18,297  17,569  16,716  15,577  16,416  11,204  8,413 | 0,465  0,48  0,726  0,688  0,687  0,74  0,718  0,696  0,656  0,687  0,5  0,389 | 4,49 | 0,58 | 0,88 | 0,35 | 0,93 | 0,95 | 0,07 | ]0,059-0,082[ |
| Training and culture | TCult1  TCult2  TCult3  TCult4  TCult5  TCult6  TCult7  TCult8  TCult9  TCult10  TCult11  TCult12  TCul13  TCult14 | 0,408  0,435  0,494  0,356  0,578  0,47  0,591  0,575  0,545  0,553  0,424  0,459  0,577  0,545 | 0,061  0,052  0,063  0,063  0,055  0,043  0,052  0,045  0,044  0,056  0,053  0,052  0,059  0,044 | 6,661  8,359  7,842  5,623  10,452  10,938  11,393  12,755  12,408  9,944  7,961  8,851  9,843  12,263 | 0,328  0,406  0,384  0,28  0,496  0,516  0,534  0,588  0,574  0,474  0,388  0,427  0,47  0,569 | 4,08 | 0,58 | 0,79 | 2,97 | 0,82 | 0,87 | 0,06 | ],054- 0,073[ |
| Extrinsic Motivation | EM1  EM2  EM3  EM4  EM5 | 0,561  0,907  0,746  0,216  0,411 | 0,053  0,058  0,064  0,07  0,063 | 10,592  15,646  11,721  3,065  6,569 | 0,53  0,856  0,593  0,156  0,331 | 4,01 | 0,77 | 0,64 | 4,16 | 0,96 | 0,97 | 0,08 | ]0,043-0,123[ |
| Self-Efficacy | SE1  SE2  SE3 | 0,353  0,792  0,727 | 0,045  0,065  0,066 | 7,809  12,211  10,987 | 0,408  0,807  0,659 | 4,15 | 0,76 | 0,65 | 1,96 | 0,99 | 0,99 | 0,04 | ]0,000-0,138[ |
| Environmental Complexity | EC1  EC2  EC3  EC4  EC5  EC6  EC7 | 0,568  0,669  0,806  0,929  0,975  0,946  0,844 | 0,055  0,06  0,054  0,063  0,059  0,061  0,058 | 10,283  11,159  14,968  14,779  16,427  15,53  14,467 | 0,486  0,53  0,667  0,659  0,717  0,694  0,659 | 3,24 | 0,92 | 0,83 | 4,27 | 0,96 | 0,97 | 0,08 | ]0,059-0,107[ |
| Expectations | E1  E2  E3  E4 | 0,52  0,53  0,751  0,568 | 0,051  0,042  0,043  0,043 | 10,16  12,543  17,557  13,326 | 0,498  0,604  0,873  0,636 | 4,48 | 0,71 | 0,77 | 0,82 | 0,99 | 1 | 0 | ]0,000-0,116[ |
| Skepticism | S1  S2  S3  S4  S5 | 0,918  0,798  1,074  0,604  0,489 | 0,067  0,067  0,071  0,075  0,076 | 13,712  11,99  15,049  8,091  6,446 | 0,674  0,589  0,743  0,412  0,334 | 2,47 | 0,96 | 0,71 | 4,04 | 0,96 | 0,97 | 0,08 | ]0,042-0,121[ |
| Overload and Fatigue | O&F1  O&F2  O&F3  O&F4  O&F5  O&F6 | 0,553  0,577  0,772  0,827  0,77  0,473 | 0,053  0,054  0,047  0,042  0,05  0,048 | 10,401  10,59  16,389  19,495  15,395  9,852 | 0,491  0,499  0,715  0,825  0,681  0,475 | 3,21 | 0,75 | 0,79 | 3,91 | 0,97 | 0,98 | 0,08 | ]0,046-0,112[ |
| Intrinsic Motivation | IM1  IM2  IM3  IM4  IM5 | 0,598  0,728  0,75  0,68  0,514 | 0,034  0,036  0,032  0,032  0,035 | 17,522  20,35  23,422  21,038  14,68 | 0,718  0,8  0,879  0,819  0,645 | 4,57 | 0,70 | 0,88 | 2,97 | 0,99 | 0,99 | 0,06 | ]0,017-0,114[ |
| Best Practices | BP1  BP2  BP3  BP4  BP5  BP6 | 1  1,359  1,655  1,449  1,224  0,59 | -  0,148  0,169  0,154  0,14  0,095 | -  9,196  9,82  9,403  8,731  6,187 | 0,478  0,674  0,824  0,71  0,605  0,359 | 3,07 | 1,01 | 0,79 | 4,09 | 0,96 | 0,97 | 0,08 | ]0,053-0,109[ |
