# Supplementary material for: An explorative approach of the InovSafeCare model for education in infection prevention and control: a cross-sectional study among undergraduate nursing students in sub-Saharan Africa
Source: Glob Health Action. 2026 Sep 15;19(1):2728504. doi: 10.1080/16549716.2026.2728504 (PMC13580379; doi:10.1080/16549716.2026.2728504)
Supplement: Supplementary file 2 [file ZGHA_A_2728504_SM8058.docx]

STROBE Statement—checklist of items that should be included in reports of observational studies

|  | Item No. | Recommendation | Page  No. | Relevant text from manuscript |
| --- | --- | --- | --- | --- |
| **Title and abstract** | 1 | (*a*) Indicate the study’s design with a commonly used term in the title or the abstract | 1 | “A cross-sectional study among undergraduate nursing students in Sub-Saharan Africa” |
|  |  | (*b*) Provide in the abstract an informative and balanced summary of what was done and what was found | 6,7 |  |
| Introduction | | | |  |
| Background/rationale | 2 | Explain the scientific background and rationale for the investigation being reported | 7 - 8 |  |
| Objectives | 3 | State specific objectives, including any prespecified hypotheses | 9 | “This study aims to explore the empirical relevance of the InovSafeCare Model in undergraduate nursing students in sub-Saharan African by assessing its theoretical structure.” |
| Methods | | | |  |
| Study design | 4 | Present key elements of study design early in the paper | 9 | “This cross-sectional observational study…” |
| Setting | 5 | Describe the setting, locations, and relevant dates, including periods of recruitment, exposure, follow-up, and data collection | 10 | “Data were collected in November 2023 from undergraduate nursing students enrolled at four sub-Saharan HEIs from two different countries (…)” |
| Participants | 6 | *Cross-sectional study*—Give the eligibility criteria, and the sources and methods of selection of participants | 10, 11 | “Students were eligible if they were 18 years old or older and enrolled in an undergraduate nursing or anesthesia program” |
| Variables | 7 | Clearly define all outcomes, exposures, predictors, potential confounders, and effect modifiers. Give diagnostic criteria, if applicable | 11, 12 | “The InovSafeCare Questionnaire was administered to assess students' opinions, perceptions, and concerns regarding theoretical and practical education in HAI-PC. In addition to measuring constructs related to healthcare-associated infections, the questionnaire includes socio-demographic questions and academic-related variables (…)” |
| Data sources/ measurement | 8* | For each variable of interest, give sources of data and details of methods of assessment (measurement). Describe comparability of assessment methods if there is more than one group | 11, 12 | “The questionnaire consists of 93 items answered on a five-point Likert scale (i.e., 1 represents strong disagreement and 5 represents strong agreement), and evaluates 14 constructs related to healthcare-associated infections (…).” |
| Bias | 9 | Describe any efforts to address potential sources of bias | 10, 11, 12 | “(…) a systematic process of cultural adaptation and translation was undertaken to ensure linguistic and contextual relevance for the target population, thereby aiming for measurement bias mitigation”; “To reduce selection bias, all eligible students present during the data collection period at the participating institutions were invited to participate (…).”; “To minimize social desirability bias and reducing the likelihood of coerced responses (…)” |
| Study size | 10 | Explain how the study size was arrived at | 10, 11 | “(…) all eligible students present during the data collection period at the participating institutions were invited to participate. The final sample (n = 486) was considered adequate for factor analysis and structural equation modeling (…)” |

Continued on next page

| Quantitative variables | 11 | Explain how quantitative variables were handled in the analyses. If applicable, describe which groupings were chosen and why | 12, 13 | “(…) descriptive analysis, including measures of central tendency, dispersion, and frequency, as well as exploratory factor analysis, and reliability analysis. Path analysis was conducted using AMOS to examine direct and indirect relationships among the studied constructs” |
| --- | --- | --- | --- | --- |
| Statistical methods | 12 | (*a*) Describe all statistical methods, including those used to control for confounding | 12 |  |
|  |  | (*b*) Describe any methods used to examine subgroups and interactions | 12-13 |  |
|  |  | (*c*) Explain how missing data were addressed | 12-13 |  |
|  |  | (*d*) *Cross-sectional study*—If applicable, describe analytical methods taking account of sampling strategy | NA |  |
|  |  | (*e*) Describe any sensitivity analyses | NA |  |
| Results | | | | |
| Participants | 13 | (a) Report numbers of individuals at each stage of study—eg numbers potentially eligible, examined for eligibility, confirmed eligible, included in the study, completing follow-up, and analysed | 14 | “(…) the study analyzed data from 486 students enrolled across the four participating institutions, non-response rate unavailable” |
|  |  | (b) Give reasons for non-participation at each stage | NA |  |
|  |  | (c) Consider use of a flow diagram |  |  |
| Descriptive data | 14 | (a) Give characteristics of study participants (eg demographic, clinical, social) and information on exposures and potential confounders | 14, 15 |  |
|  |  | (b) Indicate number of participants with missing data for each variable of interest | 14 | “The dataset contained no missing values. Therefore, all analyses were conducted using complete-case data.” |
| Outcome data | 15 | *Cross-sectional study—*Report numbers of outcome events or summary measures | 15-18 |  |
| Main results | 16 | (*a*) Give unadjusted estimates and, if applicable, confounder-adjusted estimates and their precision (eg, 95% confidence interval). Make clear which confounders were adjusted for and why they were included | 18-20 | “Relationships between constructs” |
|  |  | (*b*) Report category boundaries when continuous variables were categorized | NA |  |
|  |  | (*c*) If relevant, consider translating estimates of relative risk into absolute risk for a meaningful time period | NA |  |

Continued on next page

| Other analyses | 17 | Report other analyses done—eg analyses of subgroups and interactions, and sensitivity analyses | NA |  |
| --- | --- | --- | --- | --- |
| Discussion | | | | |
| Key results | 18 | Summarise key results with reference to study objectives | 20 | “(…)The findings underscore the complex interplay between educational strategies, individual perceptions, and contextual challenges in shaping adherence to HAI-PC measures (…)” |
| Limitations | 19 | Discuss limitations of the study, taking into account sources of potential bias or imprecision. Discuss both direction and magnitude of any potential bias | 24, 25 | “While this study provides valuable insights into the mechanisms influencing adherence to HAI-PC best practices, several limitations should be considered (…)” |
| Interpretation | 20 | Give a cautious overall interpretation of results considering objectives, limitations, multiplicity of analyses, results from similar studies, and other relevant evidence | 21-24 |  |
| Generalisability | 21 | Discuss the generalisability (external validity) of the study results | 25, 26 |  |
| Other information | |  | | |
| Funding | 22 | Give the source of funding and the role of the funders for the present study and, if applicable, for the original study on which the present article is based | 32 |  |
